# Fractional excretion of sodium and fractional excretion of urea in differentiating prerenal from renal azotemia complicating circulatory shock

**DOI:** 10.1186/2197-425X-3-S1-A262

**Published:** 2015-10-01

**Authors:** HM Sherif, AR Yassin, AY Mousa, A Esmat

**Affiliations:** Faculty of Medicine, Cairo University, Critical Care Medicine, Cairo, Egypt

## Background

Fractional excretion of sodium (FENa) is used to differentiate renal from prerenal azotemia. However, many drugs and medical problems affect the sodium (Na^+^) handling in the kidney. On the other hand, the fractional excretion of urea (FEurea) is mainly dependent on passive forces so that it is less influenced by the diuretic therapy.

## Objective

Comparison between FENa and FEurea in differentiating renal from prerenal azotemia complicating circulatory shock, and the effect of diuretics on their handling.

## Methods

Both serum FENa & FEurea were measured in 40 patients (pts) with acute kidney injury (AKI) complicating circulatory shock during their stay in ICU. All patients were divided into 26 pts (mean: 60.0 ± 15.15 years) with prerenal azotemia (group-1) and 14 pts (mean: 56.29 ± 19.5 years) with renal azotemia (group-2). Group-1 was subdivided into 12 pts (group-1a) who didn't receive diuretics 24 hours before the sampling process and 14 pts (group-1b) who received diuretics during their stay in ICU. Acute kidney injury was diagnosed according to the RIFLE-criteria (Risk, Injury, Failure, Loss and End-stage Kidney) in oliguric patients.

## Results

Both FENa and EFurea showed significantly increased incremental values with higher RIFLE-criteria from mild to severe AKI. Compared to pts of group-2, pts of group-1 showed significantly lower FENa (0.99 ± 0.66 and 2.57 ± 1.73, P < 0.05), and significantly lower FEurea (29.7 ± 7.6 and 43.7 ± 15.4, *P* < 0.001), respectively. For differentiating renal from prerenal azotemia; compared to FENa, FEurea showed better sensitivity (78.1 % vs. 71.4%) and specificity (88.5% vs. 69.4%) respectively. Moreover, compared to pts of group-1a with those of group-1b, FEurea were not significantly affected by the use of diuretics; sensitivity (78% vs. 78%) and specificity (92% vs. 88%), respectively. As a predictor of differentiation, the cutoff value of FEurea was 34.12% (*P* < 0.001) and the cutoff value of FENa was 1.1% (*P* < 0.05).

## Conclusions

FEurea is more sensitive, specific and less affected by the use of diuretics than FENa in differentiating renal from prerenal azotemia in patients with AKI complicating circulatory shock.

